# Body Self-Perception and Sense of Agency: a systematic review of literature

**DOI:** 10.1192/j.eurpsy.2022.2261

**Published:** 2022-09-01

**Authors:** S. Danilo, L. Virdia, G. Rogier, S. Beomonte Zobel, R. Cavalli, C. Civilla, P. Velotti

**Affiliations:** 1 University of Rome Sapienza, Dynamic And Clinical Psychology And Health, Rome, Italy; 2 University of Genoa, Department Of Educational Sciences, Genoa, Italy

**Keywords:** Body Self-Perception, Sense of Agency, literature, review

## Abstract

**Introduction:**

The sense of agency concerns the experience of controlling our actions, referring both to superior and lower levels of psychological functioning that are also related to the physical dimension of agency. Diverse clinical conditions affect the awareness of being a bodily and agentive self.

**Objectives:**

The aim of this systematic review was to provide a comprehensive evaluation of the relationship between individual sense of agency and body perception.

**Methods:**

PsycINFO, Psycharticle, Medline, Web of Science and Scopus were systematically searched for articles published until 19/10/2020.

**Results:**

After removing duplicates, a total of 2.556 records was screened. Fifteen articles were selected based on the inclusion criteria fixed for the systematic review.

**Conclusions:**

In the last decades, this line of research seems to attract a growing number of studies.

However, these studies are affected by a great heterogeneity in the investigation of both constructs.

Body perception and sense agency were operationalized across research in diverse ways, making the evaluation of the relationship between them very difficult. In addition, there is a paucity of studies investigating the relationship between body perception and agency among individuals suffering from specific psychopathology or physical diseases. According to literature review, the main objective for future research is to develop more robust approaches to estimate the variability of these constructs such as their relationship.

**Disclosure:**

No significant relationships.

